# LC–DAD–MS Phenolic Characterisation of Six Invasive Plant Species in Croatia and Determination of Their Antimicrobial and Cytotoxic Activity

**DOI:** 10.3390/plants11050596

**Published:** 2022-02-23

**Authors:** Danijela Poljuha, Barbara Sladonja, Ivana Šola, Mateja Šenica, Mirela Uzelac, Robert Veberič, Metka Hudina, Ibukun Michael Famuyide, Jacobus N. Eloff, Maja Mikulic-Petkovsek

**Affiliations:** 1 Department of Agriculture and Nutrition, Institute of Agriculture and Tourism, Karla Huguesa 8, 52440 Poreč, Croatia; danijela@iptpo.hr (D.P.); mirela@iptpo.hr (M.U.); 2 Department of Biology, Faculty of Science, University of Zagreb, Horvatovac 102a, 10000 Zagreb, Croatia; ivana.sola@biol.pmf.hr; 3 Department of Agronomy, Biotechnical Faculty, University of Ljubljana, Jamnikarjeva 101, 1000 Ljubljana, Slovenia; mateja.senica@bf.uni-lj.si (M.Š.); robert.veberic@bf.uni-lj.si (R.V.); metka.hudina@bf.uni-lj.si (M.H.); maja.mikulic-petkovsek@bf.uni-lj.si (M.M.-P.); 4 Phytomedicine Programme, Paraclinical Sciences Department, University of Pretoria, P/Bag X04, Onderstepoort, Pretoria 0110, Gauteng, South Africa; adeyerimi@gmail.com (I.M.F.); kobus.eloff@up.ac.za (J.N.E.)

**Keywords:** antimicrobial, cytotoxicity, ecosystem services, invasive plant species, phenolics, phytopharmaceuticals

## Abstract

Invasive plants’ phytochemicals are important for their invasiveness, enabling them to spread in new environments. However, these chemicals could offer many pharmaceutical compounds or active ingredients for herbal preparations. This study provides the first LC–MS phytochemical screening of six invasive alien plant species (IAPS) in the Istria region (Croatia): *Ailanthus altissima*, *Ambrosia artemisiifolia*, *Conyza canadensis*, *Dittrichia viscosa*, *Erigeron annuus*, and *Xanthium strumarium*. The study aims to identify and quantify the phenolic content of their leaf extracts and assess their antimicrobial and cytotoxic potential. A total of 32 species-specific compounds were recorded. Neochlorogenic, chlorogenic, and 5-*p*-coumaroylquinic acids, quercetin-3-glucoside, and kaempferol hexoside were detected in all the tested IAPS. Hydroxycinnamic acid derivatives were the main components in all the tested IAPS, except in *E. annuus,* where flavanones dominated with a share of 70%. *X. strumarium* extract had the best activity against the tested bacteria, with an average MIC value of 0.11 mg/mL, while *A. altissima* and *X. strumarium* extracts had the best activity against the tested fungi, with an average MIC value of 0.21 mg/mL in both cases. All the plant extracts studied, except *X. strumarium*, were less cytotoxic than the positive control. The results provided additional information on the phytochemical properties of IAPS and their potential for use as antimicrobial agents.

## 1. Introduction

Although invasive alien plant species (IAPS) impacts are unpredictable, they all have mechanisms that contribute to their invasiveness. They often include direct competition, indirect competition mediated by herbivores or changes in the soil community, and interference competition via allelopathy [1]. Successful alien species often owe their capability to repel native enemies to novel biochemistry [2]. Previous comparative metabolomics studies on invasive species showed that successful invasive alien plants have more total and more unique metabolites than native ones [3]. Phytochemical uniqueness appeared to be very important in the invasion ability of alien plants [4,5,6]. Therefore, phytochemicals from alien invasive species may be a leading cause of environmentally harmful effects.

On the other side, some invasive species have compounds potentially useful to humans and could provide valuable ecosystem services [7]. Many plant species possess antifungal and antibacterial activity [8,9,10]. Antimicrobial agents produced by plants’ specialised metabolism serve as their natural protective mechanism, increasing their competitiveness. 

Thus, plant extracts or plant specialised metabolites that are not toxic and are specific in their action are considered possible candidates for pharmaceuticals. They could also be active ingredients of natural preparations, such as pesticides and herbicides in organic crop production. Meela et al. [10] argued that fungal infections might control plant distribution, and the success of invasive plant species may be due to their antifungal activity, which they found in seven invasive species. This may help fend off fungal pathogens or disturb established symbiotic relationships in their new habitat. Eloff et al. [11] showed that an easy solvent–solvent fractionation process of acetone leaf extracts of the invasive *Melianthus comosus* Vahl led to a product with much higher activity than commercial fungicides. The activity of this product in field trials was also much better than that of the commercial product. They also stated that toxic antifungal extracts might be useful in the floricultural industry. 

Up to date, there have been very little literature data on the phytochemical potential of IAPS or their exploration in Croatia [9,12,13,14]. For our study, we selected six IAPS, all included in the list of the most relevant invasive plant species of Croatia [15] and widely present in the Istria region (the peninsula in west Croatia). Previous phytochemical studies revealed the presence of different bioactive compounds in those species: *Ailanthus altissima* (Mill.) Swingle [16], *Ambrosia artemisiifolia* L. [17], *Conyza canadensis* (L.) Cronquist [18], *Dittrichia viscosa* (L.) Greuter [19], *Xanthium strumarium* L. [20], and *Erigeron annuus* (L.) Pers. [21]. The extracts of selected plant species are used in traditional medicine in China and India, and their antimicrobial properties have been reported [22,23,24,25,26,27]. Our previous research [9] showed that *A. altissima* from Croatia could be a valuable resource for antimicrobial activity since its leaf extracts were as effective against *Escherichia coli* and *Candida albicans* as standard antibiotics. 

Phenolic compounds in plants are generally one of the most significant bioactive groups, playing an essential role in their development and defence against infection and injury [28]. Higher concentrations of phenolic compounds in invasive plants may provide a competitive advantage over native species [6]. Still, the role and function of high phenolic diversity remain to be elucidated. 

The phytochemical characterisation presented in this study is one step forward in this task. This knowledge is also crucial to define the possible application areas for the rational use of these species. 

In our study, we identified and quantified phenolics in leaves of six IAPS from the territory of Istria (Croatia) for the first time (except for *A. altissima*) and tested the antibacterial, antifungal, and cytotoxic activities of their extracts as a basis for new possible ecosystem services.

## 2. Results and Discussion

### 2.1. Extraction Yield and Phenolic Concentration

The extraction technique’s efficacy is manifested in the yield of an extract with maintained functional properties. We prepared two types of extracts from six selected IAPS. We used methanol as an extractant to prepare phenolic extracts and acetone for the bioassays (cytotoxicity and antimicrobial testing) due to its lower toxicity to fungi [29] and based on the comparison of different extractants [30,31]. We obtained the highest phenolic concentration from *D. viscosa* (68 mg/g), *E. annuus* (46 mg/g), and *A. altissima* (42 mg/g), followed by *X. strumarium* (29 mg/g), *A. artemisiifolia* (15 mg/g), and *C. canadensis* (13 mg/g) (Table 1). 

When we used acetone as an extractant, we obtained the highest yield from *A. artemisiifolia* (101 mg/g), followed by *E. annuus* (66 mg/g), *D. viscosa* (63 mg/g), *X. strumarium* (57 mg/g), *C. canadensis* (47 mg/g), and finally *A. altissima* with the lowest extraction yield of 42 mg/g (Table 2 and Table 3).

### 2.2. LC–DAD–MS Identification and Quantification of Phenolic Compounds

The highest number of individual compounds was identified in *A. artemisiifolia* (30), followed by *E. annuus* (26), *X. strumarium* (22), *D. viscosa* (22), *A. altissima* (21), and *C. canadensis* (18) (Supplementary Table S1). The identified compounds belong to the groups of hydroxycinnamic and hydroxybenzoic acid derivatives, flavones, flavanols, flavonols, flavanones, ellagitannins, and gallotannins (Supplementary Table S1). As expected, the highest number of individual compounds were identified in the groups of flavonols and hydroxycinnamic acid derivatives because it is known that flavonols such as quercetin and kaempferol [32], as well as acids such as caffeic, *p*-coumaric, and ferulic, predominate in Asteraceae [33] and *A. altissima* [9,34]. The compounds recorded in each species were neochlorogenic, chlorogenic, and 5-*p*-coumaroylquinic acids, quercetin-3-glucoside, and kaempferol hexoside. This is in accordance with other studies. Neochlorogenic acid has been recognised as the main antioxidant component of one of the most invasive plant species, *Polygonum cuspidatum* Siebold & Zucc. [35]. Chlorogenic acid is one of the major phenolic components in *Lonicera maackii* (Rupr.) Maxim., an invasive shrub in North America [36]. In [37], the invasive species *Tithonia diversifolia* (Hemsl.) A.Gray also contained a significant amount of chlorogenic acids. Very recently, 5-*p*-coumaroylquinic acid was recorded in phytotoxic *Lolium multiflorum* Lam. [38], and quercetin glycosides were redundantly present in allelopathic *Trigonella foenum graecum* L. [39]. Further studies are needed to determine whether these compounds may have an effect on the invasive ability of invasive species. The proposed activities of these compounds range from antimicrobial activity, drought, or heat resistance to allelopathy or others. As shown in Supplementary Table S1, we also detected 32 compounds that were present in only one of the tested species, according to the following representation: 9 in *A. altissima* (4-*p*-coumaroylquinic acid, ellagic acid pentoside, apigenin hexoside, quercetin galloyl hexosides 1 and 2, quercetin acetyl hexoside, kaempferol acetyl hexoside 2, vescalagin isomer, and digalloyl-HHDP-hexoside isomer), 7 in *A. artemisiifolia* (caftaric acids 1 and 2, caffeic acid, caffeic acid derivative 2, isorhamnetin-acetyl-hexoside, isorhamnetin-3-rutinoside, and gallic acid), 6 in *E. annuus* (ellagic acid rhamnoside, quercetin-3-rhamnoside, kaempferol-3-rutinoside, kaempferol-glucuronyl-hexoside, myricetin-3-glucuronide, and naringenin hexoside 2), 5 in *X. strumarium* (apigenin, procyanidin dimer, epicatechin, laricitrin, and syringetin), 4 in *D. viscosa* (dicaffeoylquinic acids 4 and 5, ellagic hexoside 2, and myricetin hexoside), and 1 in *C. canadensis* (ellagic acid). The fact that 9 out of 21 identified compounds in *A. altissima* were present in this species only was expected since only this species belongs to the small family Simaroubaceae, while others are from the ubiquitously present Asteraceae family. While 5-*p*-coumaroylquinic acid was present in all the tested species except *E. annuus*, and 3-*p*-coumaroylquinic in all except *X. strumarium*, 4-*p*-coumaroylquinic acid was detected in *A. altissima* only, so we assume that the biosynthesis of this isomer of chlorogenic acid is more constrained. As well, 5-*p*-coumaroylquinic acid has been detected in another invasive species, for example, in *Ageratina adenophora* (Spreng.) R.M.King & H.Rob. [40]. All three *p*-coumaroylquinic acid isomers were recorded in *A. altissima* only (Supplementary Table S1). The combination of three isomers can be found in the leaves of *Vaccinium uliginosum* L. (Ericaceae) as well [41]. In *A. altissima* only, we also recorded digalloyl-HHDP (6,6′-dicarbonyl-2,2′,3,3′,4,4′-hexahydroxybiphenyl)-hexoside and vescalagin. These compounds are present in wild *Fragaria vesca* L. fruits [42] and *Alchemilla vulgaris* L. and *Alchemilla mollis* (Buser) Rothm. [43]. 

Only *A. altissima* and *A. artemisiifolia* contained gallotannins, namely, digalloyl-HHDP-hexoside isomer and gallic acid, respectively (Table 1). Gallic acid occurs widely in plants and has antioxidant activities. Plants contain many antioxidant compounds, especially in leaves, because photosynthesis generates free radicals that have to be neutralised. Gallic acid has previously been identified in the invasive shrub *Rhododendron ponticum* [44]. Moreover, it was recorded that it had a dose-dependent inhibitory effect on the seed germination and seedling growth of cucumber (*Cucumis sativus* L.) [45]. Furthermore, it has been detected as one of the compounds responsible for the allelopathy of *Polygonella myriophylla* [46]. However, some data show that this acid alone is not sufficient for the successful invasion of *Phragmites australis* [47]. Therefore, it might be that gallic acid contributes to plant allelopathy in combination with other compounds. Hydroxycinnamic and hydroxybenzoic acid derivatives were quantitatively the main components of the phenolic fraction in all the tested plant extracts, except *E. annuus*, where flavanones dominated with a share of 70% (Table 1, Supplementary Figure S1). The abundant representation of flavanones in *E. annuus* has already been documented [48]. Hydroxycinnamic acid derivatives were significantly more present in *D. viscosa* and *X. strumarium* than in other species (Table 1).

As shown in Table 1, the highest concentration of total identified phenolic compounds was recorded in the extracts of *D. viscosa* (67.78 ± 0.57 mg/g DW). Moreover, among the identified compounds, 93% belonged to the groups of hydroxycinnamic and hydroxybenzoic acid derivatives. The predominant ones were caffeic acid and quercetin derivatives, similar to the Tunisian ecotype [49]. At the same time, *A. artemisiifolia* and *C. canadensis* extracts contained significantly lower concentrations (15.00 ± 0.42 and 13.00 ± 2.90 mg/g DW, respectively) of total identified phenolic compounds. *C. canadensis* had the lowest concentration of total identified phenolic compounds (Table 1, Supplementary Table S1). According to Queiroz et al. [50], this species contains acetylenes, which could be responsible for its allelopathic activity. The highest concentration of a single group of compounds we recorded in *E. annuus*—naringenin hexosides, at a concentration of 32.07 ± 3.01 mg/g DW (Table 1). Moreover, it is only in this species that we have recorded these compounds. Naringenin-6,8-di-C-hexoside has already been detected in the wild plants *Lathyrus aureus* (Steven) D.Brandza and *Lathyrus pratensis* L., which have been recognised as new sources of biologically active compounds [51].

Regarding the flavonoids, all the tested species contained quercetin and kaempferol derivatives. *A. altissima*, *A. artemisiifolia*, *D. viscosa*, and *X. strumarium* had more quercetin derivatives, while in *C. canadensis* and *E. annuus*, kaempferol derivatives dominated. Isorhamnetin derivatives were detected in *A. artemisiifolia* and *D. viscosa*, and myricetin derivatives in *D. viscosa* and *E. annuus*. The two species that contained all the four flavonol derivatives were *D. viscosa* and *E. annuus*. Flavanols were detected in *X. strumarium* only, and ellagitannin vescalagin in *A. altissima* only. The flavanones naringenin-hexosides were present in *C. canadensis* and *E. annuus*, the flavones apigenin and apigenin-hexoside in *X. strumarium* and *A. altissima*, respectively. The gallotannins digalloyl-hexahydroxydiphenoyl (HHDP)-hexoside isomer and gallic acid were present in *A. altissima* and *A. artemisiifolia,* respectively. Digalloyl-HHDP-glucoside has already been identified in flowers of pomegranate (*Punica granatum* L.) [52].

As shown in Table 1, we recorded the highest concentration of a particular phenolic group in *E. annuus* for flavanones (32.07 ± 3.01 mg/g DW, in particular, naringenin hexosides 1 and 2) and in *D. viscosa* for hydroxybenzoic acid derivatives (31.63 ± 0.53 mg/g DW, ellagic acid hexosides 1 and 2) and hydroxycinnamic acid derivatives (31.08 ± 0.11 mg/g DW, nine different types) (Supplementary Table S1). At the same time, these two species also had the highest concentration of all the identified compounds. Among the hydroxybenzoic acid derivatives, in all the species, the predominant were caffeic acid derivatives with even 31.02 ± 0.01 mg/g DW in *D. viscosa* (Table 1). This species also contained the highest concentration of hydroxybenzoic acid derivatives, in particular, ellagic acid hexosides at a concentration of 31.63 ± 0.53 mg/g DW.

Statistical comparison of the phenolic groups’ concentrations between the species revealed the following: *D. viscosa* and *X. strumarium* were dominant over the other species in hydroxycinnamic acid derivative concentration, *D. viscosa* had a higher concentration of hydroxybenzoic acid derivatives than other species, and *E. annuus* was predominant in flavanone concentration (Table 1). *A. altissima* had significantly higher flavones and tannin concentrations than the other species. 

In all the species, hydroxycinnamic and hydroxybenzoic acid derivatives dominated, with the share in total identified phenolic compounds between 62% (*A. altissima*) and 93% (*D. viscosa*). The only exception was *E. annuus*, where the predominant compounds were flavanones with a share of 70% (Table 1, Supplementary Figure S1). Caftaric acid was recorded only in *A. artemisiifolia*. This type of acid can otherwise be found in the leaf and flower of *Lapsana communis* L. subsp. *communis* (Asteraceae), a plant used in folk medicine as a poultice against chapped nipples or hands [53]. The isomer of catechin, epicatechin, was detected in *X. strumarium.* It has already been shown previously that catechin contributes to the invasiveness of *Centaurea stoebe* Tausch [54] and *Rhododendron ponticum* L. [44]. Laricitrin, which we also recorded only in *X. strumarium*, was recently detected in the invasive alien species *Rhododendron ponticum* [44], which suggests the implication of this compound in the plants’ invasion process.

### 2.3. Antimicrobial Activity and Bioautography

The ethyl acetate/methanol/water (40:5.4:5) (EMW) eluent system defines separated components in almost all plant species’ crude extracts; however, it moves most of the components up close to the solvent front, making it difficult to notice good separation. Most of the compounds in the extracts were of intermediate polarity or nonpolar (Supplementary Figure S2a). The bioautography assay showed that at least one compound from each plant extract was active against the tested bacteria with a relatively clear zone on TLC bioautograms, indicating growth inhibition (Supplementary Figure S2b–d), except those from *A. artemisiifolia* and *C. canadensis* against *Pseudomonas aeruginosa* (Supplementary Figure S2d). This suggests a higher resistance of *P. aeruginosa* to *A. artemisiifolia* and *C. canadensis* bioactive compounds. 

The antimicrobial activity of plant extracts was classified based on the MIC values as follows: outstanding (0.02 mg/mL and below), excellent (0.021–0.04 mg/mL), very good (0.041–0.08 mg/mL), good (0.08–0.16 mg/mL), average activity (0.161–0.32 mg/mL), and weak (>0.32 mg/mL) [55].

The extract of *X. strumarium* had good activity against three of the four tested bacteria, with an average MIC value of 0.11 mg/mL, which is a promising antibacterial activity. The extracts of *D. viscosa* and *A. altissima* had an average activity with an average MIC value of 0.21 and 0.22, respectively (Table 2). *D. viscosa* had the highest concentration of total identified compounds (67.78 ± 0.57 mg/g DW), and especially hydroxycinnamic and hydroxybenzoic acid derivatives (31.08 ± 0.11 mg/g DW and 31.63 ± 0.53 mg/g DW, respectively) (Table 1). Since this species also showed the lowest MIC value against *E. coli* (Table 2), we hypothesise that activity against this bacteria strain might have been mediated via the identified hydroxycinnamic and hydroxybenzoic acid derivatives. The highest activity of all plant extracts was obtained against *Enterococcus faecalis*, with each observed MIC value below 0.1 mg/mL. The lowest activity was obtained against *Staphylococcus aureus* and *P. aeruginosa*, with average MIC values of 0.69 and 0.64 mg/mL, respectively (Table 2). Many of these activities are below values considered interesting for developing new antibiotics (MIC <1 mg/mL) [56]. Still, they could be very useful when used in the organic production of plant products if they are not edible because a higher concentration can be applied. 

In addition to the MIC values, we also determined the total antibacterial activity (TAA) value, which takes into account the mass extracted from each species to compare plant extracts. TAA value (obtained by dividing yield by the MIC and expressed in ml/g) shows the largest volume to which the biologically active compounds present in 1 g of extract can be diluted and still inhibit the growth of the test organism [56]. *X. strumarium* and *A. artemisiifolia* extracts had the highest average TAA of 775 and 624 mL/g, respectively (Table 2). The values varied between 248 and 1425 mL/g with *X. strumarium* and between 160 and 1683 mL/g with *A. artemisiifolia* extract against different bacteria. This is an important parameter to compare the efficacy of different plants. Thus, 1 g of *X. strumarium* extract can be diluted to 1425 mL, and 1 g of *A. artemisiifolia* extract to even 1683 mL, and will still inhibit the growth of *E. faecalis*. In this particular case, the extract of *A. artemisiifolia* with the highest TAA of 1683 mL/g was the most effective against *E. faecalis*.

The cytotoxicity was determined using an in vitro assay against Vero monkey kidney cells. The LC_50_ and the selectivity index (SI) values were calculated (Table 2). The LC_50_ values ranged from 0.002 to 0.13 mg/ ml. Extracts of *A. altissima* and *C. canadensis* had the highest LC_50_ (lowest toxicity) of 0.17 mg/mL, both, while the extract of *X. strumarium* was the most toxic (0.001 mg/mL) to the cells. In classifying the in vitro cytotoxicity of plant extracts, values below 20 µg/mL are considered highly cytotoxic [57]. However, caution must be taken in making conclusions based on in vitro cytotoxic data only, because in vitro toxicity data may not always agree with in vivo toxicity test owing to a number of factors, such as gut interactions, solubility, bioavailability, and other pharmacokinetic/dynamic parameters. Therefore, further toxicity testing in in vivo systems will be useful. The selectivity index (SI) is a ratio that measures the window between cytotoxicity and antimicrobial (antibacterial) activity by dividing the given LC_50_ by the MIC value. The higher the SI ratio, the theoretically more effective and safe an extract would be during in vivo treatment for a given bacterial (fungal) infection, and the more likely it is that the activity is not due to a general metabolic toxin. Extracts of *A. altissima*, *C. canadensis*, *E. annuus*, and *D. viscosa* had SI values higher than 1 (Table 2) against *E. faecalis*.

The extracts of *A. altissima* and *X. strumarium* had the highest average activity against two of the three tested fungi, with an average MIC value of 0.21 mg/mL (Table 3). *A. altissima* showed the greatest variety of compounds (most specific ones) and also the least MIC value toward *A. fumigatus*. Hence, we hypothesise that some or a combination of some compounds could be responsible for higher toxicity of *A. altissima* extract to *A. fumigatus*. The highest activity of all plant extracts was obtained against *C. albicans*, with an average MIC value of 0.20 mg/mL. *A. fumigatus* was the most resistant fungus against the plant extracts with an average MIC value of 0.69 mg/mL. In comparison with amphotericin B, used as a positive control, with an average MIC value of 8 × 10^−3^ mg/mL, tested extracts were much less active.

The *X. strumarium* and *A. artemisiifolia* extracts had the highest average TAA of 284 and 296 mL/g, respectively (Table 3). The highest value for *X. strumarium* extract was 365 mL/g, which means that the yield from 1 g of *X. strumarium* extract can be diluted to 365 mL and will still inhibit the growth of *C. neoformans*. *A. artemisiifolia* had the highest TAA value (646 mL/g) against *C. albicans*.

None of the tested extracts had an SI ratio greater than 1, implying that the antifungal activity of the plant extract is probably due to a general metabolic toxic effect (Table 3). It should be noted, however, that in vitro toxicity results may not always predict the toxicity behaviour of extracts when tested in in vivo systems due to pharmacokinetic and pharmacodynamic interactions within the body [58].

## 3. Materials and Methods

### 3.1. Plant Material

The leaves of six invasive species widespread in Croatia were used in this study: *Ailanthus altissima* (Mill.) Swingle (Simaroubaceae), *Ambrosia artemisiifolia* L. (Asteraceae), *Conyza canadensis* (L.) Cronquist (Asteraceae), *Dittrichia viscosa* (L.) Greuter (Asteraceae), *Erigeron annuus* (L.) Pers. (Asteraceae), and *Xanthium strumarium* L. (Asteraceae). The official blacklist of the invasive species of Croatia, according to the law on the introduction, prevention, and spread of invasive alien species and management [59], has not been delivered yet. However, Nikolić et al. [14] published a list of 70 most relevant invasive plant species of Croatia, which contains all the species involved in this research. Moreover, all of them, except *D. viscosa,* are included in the Global Register of Introduced and Invasive Species [60].

**Figure 1:**
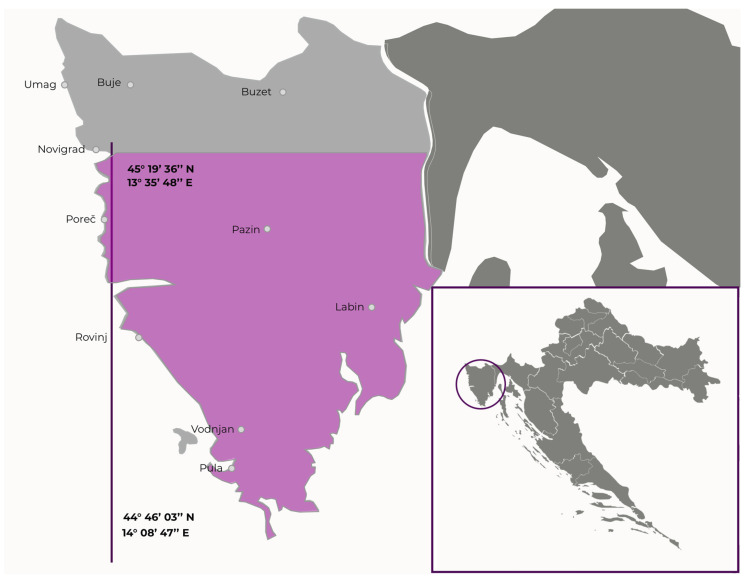
Location (geographic coordinates) in the Istria region where the six invasive alien plant species were collected. The location of the Istria Region in Croatia is given in the bottom right.

The plants were collected in the Istria region (Figure 1). Their identities were established by Istrian Botanic Society botanists and confirmed by comparison with the literature. For each species, plant material from different locations (latitude from 44°46′03″ N to 45°19′36″ N, longitude from 13°35′48″ E to 14°08′47″ E) was collected in the optimal maturity stage for determination, from June to August 2018. Approximately 250 g of plant material from every location was collected and pooled. For phenolic profiling, plant material was freeze-dried and stored in the dark at room temperature until analysis. For antimicrobial assays, plants were air-dried in the shade and stored at room temperature.

### 3.2. Extraction Procedures

The extraction of phenolic compounds for LC–MS identification and quantification was performed following the slightly modified procedure of Mikulic-Petkovsek et al. [61]. A hundred milligram of dried plant tissue was extracted with 6 mL of 70% methanol containing 3% (*v/v*) formic acid in a cooled ultrasonic bath for 60 min. Extracts were centrifuged for 10 min at 10,000× *g* and filtered through 20 µm polytetrafluoroethylene (PTFE) filters (Macherey-Nagel, Düren, Germany). Identification of individual phenolic compounds was qualitatively achieved using the method of external standards and quantitatively comparing peak area on chromatograms of samples with those of diluted standard solutions. The external standards used in this study were caffeic acid, apigenin-7-glucoside, ferulic acid, quercetin-3-*O*-rhamnoside, neochlorogenic (3-caffeoylquinic) acid, naringenin, ellagic acid, gallic acid, chlorogenic acid, and rutin (quercetin-3-*O*-rutinoside) from Sigma-Aldrich (St. Louis, MI, USA) Chemie; (-)epicatechin, quercetin-3-*O*-galactoside, quercetin-3-*O*-glucoside, *p*-coumaric acid, procyanidin B1, and kaempferol-*O*-glucoside from Fluka Chemie; quercetin-3-*O*-xyloside and quercetin-3-*O*-arabinopyranoside from Apin Chemicals (Abingdon, UK); and isorhamnetin-3-*O*-glucoside from Extrasynthese (Genay, France).

Acetone was used as an extractant in bioassays (cytotoxicity and antimicrobial activities). The choice of extractant was based on its efficacy in the quantity and diversity of compounds extracted, safety, low toxicity, and ease of removal [62]. The mixture of finely ground dry leaf samples (2 g) and acetone (20 mL) was sonicated for 20 min, vigorously shaken, and then centrifuged for 10 min at 4000× *g* (Hettich Centrifuge, Rotofix 32 A, Labotec, Johannesburg, South Africa). The supernatant was filtered through a filter paper (Whatman No. 1) and concentrated by drying under a stream of cold air in preweighed glass vials. The yield was obtained by dividing the mass extracted by the initial mass. A stock solution (10 mg/mL) in acetone was prepared and used in the assays.

### 3.3. Determination of Individual Phenolic Compounds Using LC–DAD–MS Analysis

Phenolic compounds were analysed on an HPLC system (Thermo Finnigan Surveyor, Thermo Scientific, San Jose, CA, USA) equipped with a DAD detector set (280, 350, and 530 nm) following the procedure of Mikulic-Petkovsek et al. [61]. A Gemini C18 (Phenomenex, Torrance, CA, USA) column (150 × 4.6 mm i.d., 3 μm) was used at 25 °C. The samples were eluted with aqueous 0.1% formic acid and 3% acetonitrile in double-distilled water (A) and 0.1% formic acid/3% double-distilled water in acetonitrile (B) according to linear gradients reported by Wang et al. [63]. The Excalibur software was used for spectral data analyses (Thermo Fisher Scientific, Waltham, MA, USA). All phenolic compounds were identified using a mass spectrometer Thermo Finnigan LCQ Deca XP Mass (Thermo Fisher Scientific, Waltham, MA, USA) with an electrospray interface (ESI) operating in negative and positive ion modes, performing analyses under the same conditions as reported by Mikulic-Petkovsek et al. [64]. Identification of the phenolic compounds was established based on their retention times and their PDA spectra in comparison with standard phenolics and based on fragmentation patterns in different MSn modes compared with literature data. Contents of phenolics were expressed in mg/g dry weight (DW) of plant material. All analyses were performed in triplicate.

### 3.4. Antimicrobial Testing

#### 3.4.1. Bioautography

Chemical constituents of the extracts were analysed by thin-layer chromatography (TLC) using aluminium-backed TLC plates (silica gel 60 F254, Merck, Kenilworth, NJ, USA). The TLC plates were developed under saturated conditions with ethyl acetate/methanol/water (40:5.4:5) (EMW) (polar/neutral) eluent systems developed in the laboratory of the Department of Paraclinical Sciences, University of Pretoria, which efficiently separate components of plant extracts [31]. To detect the separated compounds, vanillin–sulphuric acid (0.1 g vanillin (Sigma^®^, Pretoria, South Africa): 28 mL methanol: 1 mL sulphuric acid) was sprayed on the chromatograms and heated at 110 °C for optimal colour development.

A bioautographic method used by Kotze and Eloff [31] was used to determine the antibacterial activity of separated compounds. TLC plates (10 cm × 10 cm) were loaded with 10 μL of a 10 mg/mL solution of each of the extracts. The prepared plates were developed in an EMW mobile system. The chromatograms were dried at room temperature under a stream of air to remove the remaining solvent. The TLC plates were sprayed with a concentrated suspension of actively growing cells of four bacterial strains before incubating at 38 °C in a chamber at 100% relative humidity. Plates were sprayed with a 2 mg/mL solution of *p*-iodonitrotetrazolium violet (Sigma). Clear zones indicated inhibition of growth on the chromatogram after incubating for 1 h.

#### 3.4.2. Antibacterial and Antifungal Activity

The minimal inhibitory concentrations (MICs) of extracts on the selected microbes were determined using the serial microdilution method in 96-well microplates previously developed by Eloff [62]. Antibacterial activity of plant extracts was tested against two Gram-positive (*Staphylococcus aureus* ATCC 29213 and *Enterococcus faecalis* ATCC 29212) and two Gram-negative (*Escherichia coli* ATCC 25922 and *Pseudomonas aeruginosa* ATCC 27853) bacterial strains. Bacteria strains were purchased from Anatech Instruments (PTY) LTD, Pretoria, South Africa. Frozen stocks of the bacteria were grown on Mueller Hinton agar (Sigma) and kept in the fridge. Prior to the assay, bacteria were grown in Mueller Hinton broth (Sigma) overnight and adjusted to a McFarland 1 standard. One hundred microlitres of each extract (10 mg/mL) dissolved in acetone was added to the first well of a round-bottomed sterile 96-well plate containing 100 µL of sterile distilled water and serially diluted (1:1). Then 100 µL of bacteria was added to the wells and incubated for 18–24 h. The bacteria were exposed to final extract concentrations of 2.5, 1.25, 0.63, 0.32, 0.16, 0.08, 0.04, and 0.02 mg/mL. Gentamicin and acetone served as positive and negative controls, respectively. Afterwards, 40 μL of *p*-iodonitrotetrazolium violet (INT; Sigma^®^) dissolved in hot sterile water was added to the wells with further incubation for 1–2 h. The MIC was determined visually as the lowest concentration inhibiting bacteria growth.

As regards antifungal activity, a slightly modified serial dilution method by Masoko et al. [65] was used to determine the MIC of extracts against fungi (*Candida albicans* ATCC 10231, *Cryptococcus neoformans* ATCC 32045, and *Aspergillus fumigatus* ATCC 204305). Fungi cultures were purchased from Anatech Instruments (PTY) LTD, Pretoria, South Africa. Fungi were maintained on Sabouraud agar (Sigma) prior to growth in Sabouraud broth (Sigma) overnight. The assay was performed as described above, except that amphotericin B served as a positive control, while INT (40 µL) was added to the wells prior to incubation for 24 h.

### 3.5. Cytotoxicity

Cytotoxicity (LC_50_) assessment was performed using the 3-(4,5-dimethylthiazol-2-yl)-2,5-diphenyltetrazolium bromide (MTT) assay on Vero African green monkey kidney cells, according to Mosmann [66], with minor modifications [67]. Cells were kept in a minimal essential medium (Highveld Biological (PTY) LTD, Johannesburg, South Africa) supplemented with 5% foetal calf serum (Adcock Ingram, South Africa) and 0.1% gentamicin (Virbac, Johannesburg, South Africa) in a 5% CO_2_ incubator. Cells were prepared from not more than 80% monolayers and seeded at a density of 1 × 10^4^ cells/ well of sterile 96-well microtiter cell culture plates and incubated at 37 °C and 5% CO_2_ in a humidified environment for 24 h. Following this, cells were exposed to extracts at varying concentrations in quadruplicate and incubated for 48 h. Doxorubicin (Pfizer Laboratories, Johannesburg, South Africa) served as a positive control, while untreated cells served as a negative control. Afterwards, the medium in each well was aspirated, and the wells were washed with PBS (Whitehead Scientific, Johannesburg, South Africa), and a fresh complete medium was added to each well. Then, 30 μL of 0.5% MTT (Sigma, Pretoria, South Africa) was added to the wells, and the plates were incubated at 37 °C for 4 h. Media in the wells were discarded, and DMSO was added to each well to solubilise the formazan crystals. The absorbance was measured using a BioTek Synergy microplate reader (Thermo Fisher Scientific, USA) at 570 nm, and LC_50_ values of the extracts were calculated. The selectivity index (SI) value for each sample was obtained by dividing the LC_50_ value by the respective MIC value (SI = LC_50_/MIC).

### 3.6. Statistical Analyses

The data were analysed in the Statgraphics Plus 4.0 program (Manugistics Inc., Rockville, MD, USA) using one-way analysis of variance (ANOVA) and LSD multiple range test (*p* ≤ 0.05).

## 4. Conclusions

The results of this study add valuable information to the existing knowledge on invasive plant species and might contribute to the use of these underutilised and undesirable species for the development of commercial natural products. All the extracts proved to be a rich source of phenolics, with very good to average antimicrobial activity against four bacterial strains and three fungi. The observed activity against fungi indicates that extracts of these plants could be useful to combat fungal infections of plants. In the cases where the selectivity index was too low, it could still be used on nonedible plants, such as flowers.

## Figures and Tables

**Table 1:** The concentrations of identified phenolics in six invasive plant species (mg/g DW ± standard error). Data are presented as a heat map in Supplementary Figure S1.

	*Ailanthus* *altissima*	*Ambrosia* *artemisiifolia*	*Conyza* *canadensis*	*Dittrichia* *viscosa*	*Erigeron* *annuus*	*Xanthium* *strumarium*	Stat. Significance
Dicaffeoylquinic acids	n.d.	3.78 ± 0.06	4.76 ± 0.52	22.36 ± 0.14	3.99 ± 0.33	11.46 ± 0.18	
3-caffeoylquinic acid	2.44 ± 0.20	0.37 ± 0.00	0.26 ± 0.02	0.24 ± 0.01	0.18 ± 0.01	0.61 ± 0.06	
4-caffeoylquinic acid	2.83 ± 0.28	0.28 ± 0.02	0.28 ± 0.03	n.d.	0.13 ± 0.02	0.20 ± 0.02	
5-caffeoylquinic acid	7.42 ± 1.11	5.04 ± 0.15	2.72 ± 0.10	8.42 ± 0.08	2.86 ± 0.21	11.25 ± 0.09	
triCQA	n.d.	n.d.	n.d.	n.d.	1.06 ± 0.04	0.83 ± 0.04	
Caffeic acid	n.d.	0.43 ± 0.00	n.d.	n.d.	n.d.	n.d.	
Other caffeic acid derivatives	n.d.	0.21 ± 0.01	n.d.	n.d.	0.15 ± 0.00	n.d.	
**Caffeic acid derivatives total**	**12.68 ± 1.62 ^b^**	**10.11 ± 0.28 ^b^**	**8.02 ± 0.77 ^b^**	**31.02 ± 0.01 ^a^**	**8.37 ± 0.72 ^b^**	**24.35 ± 0.81 ^a^**	***
**Caftaric acids**	**n.d.**	**3.72 ± 0.09**	**n.d.**	**n.d.**	**n.d.**	**n.d.**	
3-*p*-coumaroylquinic acid	0.09 ± 0.02	0.03 ± 0.01	0.04 ± 0.01	0.03 ± 0.01	0.05 ± 0.00	n.d.	
4-*p*-coumaroylquinic acid	0.24 ± 0.05	n.d.	n.d.	n.d.	n.d.	n.d.	
5-*p*-coumaroylquinic acid	0.68 ± 0.14	0.11 ± 0.01	0.04 ± 0.00	0.03 ± 0.01	n.d.	0.04 ± 0.00	
***p*-coumaric acid derivatives**	**1.02 ± 0.19 ^a^**	**0.14 ± 0.01 ^b^**	**0.09 ± 0.01 ^b^**	**0.07 ± 0.01 ^b^**	**0.05 ± 0.00 ^b^**	**0.04 ± 0.00 ^b^**	**
3-feruloylquinic acid	0.09 ± 0.01	0.03 ± 0.00	0.03 ± 0.01	n.d.	0.01 ± 0.00	0.01 ± 0.00	
5-feruloylquinic acid	n.d.	0.31 ± 0.02	n.d.	n.d.	n.d.	0.34 ± 0.04	
**Ferulic acid derivatives**	**0.09 ± 0.01 ^b^**	**0.35 ± 0.01 ^a^**	**0.03 ± 0.00 ^c^**	**n.d.**	**0.01 ± 0.00 ^c^**	**0.35 ± 0.04 ^a^**	***
**Hydroxycinnamic acid derivatives**	**13.79 ± 1.82 ^b^**	**10.60 ± 0.29 ^b^**	**8.14 ± 0.78 ^b^**	**31.08 ± 0.11 ^a^**	**8.21 ± 0.73 ^b^**	**24.39 ± 0.42 ^a^**	***
Ellagic acid	n.d.	n.d.	3.24 ± 1.61	n.d.	n.d.	n.d.	
Ellagic acid pentoside	12.13 ± 2.31	n.d.	n.d.	n.d.	n.d.	n.d.	
Ellagic acid hexosides	n.d.	n.d.	n.d.	31.63 ± 0.43	1.07 ± 0.18	n.d.	
Ellagic acid rhamnoside	n.d.	n.d.	n.d.	n.d.	0.07 ± 0.01	n.d.	
**Hydroxybenzoic acid derivatives**	**12.13 ± 2.39 ^b^**	n.d.	**3.24 ± 1.97 ^bc^**	**31.63 ± 0.53 ^a^**	**1.14 ± 0.23 ^c^**	n.d.	***
Apigenin	n.d.	n.d.	n.d.	n.d.	n.d.	0.05 ± 0.01	
Apigenin hexoside	0.87 ± 0.07	n.d.	n.d.	n.d.	n.d.	n.d.	
**Flavones**	**0.87 ± 0.07 ^a^**	n.d.	n.d.	n.d.	n.d.	**0.05 ± 0.01 ^b^**	***
Procyanidin dimer	n.d.	n.d.	n.d.	n.d.	n.d.	1.19 ± 0.09	
Epicatechin	n.d.	n.d.	n.d.	n.d.	n.d.	0.02 ± 0.00	
**Flavanols**	n.d.	n.d.	n.d.	n.d.	n.d.	**1.21 ± 0.11**	
Quercetin-dihexoside	n.d.	1.19 ± 0.06	0.04 ± 0.01	n.d.	0.15 ± 0.02	n.d.	
Quercetin-galloyl-hexosides	0.69 ± 0.07	n.d.	n.d.	n.d.	n.d.	n.d.	
Quercetin-3-galactoside	2.81 ± 0.28	0.45 ± 0.01	n.d.	0.25 ± 0.10	n.d.	n.d.	
Quercetin-3-glucoside	0.56 ± 0.05	0.10 ± 0.00	0.05 ± 0.01	0.11 ± 0.01	0.01 ± 0.00	0.34 ± 0.00	***
Quercetin-3-rutinoside	n.d.	0.24 ± 0.00	n.d.	0.25 ± 0.01	n.d.	0.10 ± 0.02	
Quercetin-3-xyloside	n.d.	0.01 ± 0.00	0.06 ± 0.01	0.08 ± 0.00	n.d.	0.05 ± 0.01	
Quercetin-3-rhamnoside	n.d.	n.d.	n.d.	n.d.	0.27 ± 0.02	n.d.	
Quercetin-3-glucuronide	n.d.	0.43 ± 0.01	0.07 ± 0.02	0.24 ± 0.00	0.08 ± 0.01	0.75 ± 0.02	
Quercetin-3-arabinopyranoside	n.d.	0.10 ± 0.00	0.10 ± 0.01	1.32 ± 0.09	n.d.	n.d.	
Quercetin-acetyl hexoside	0.13 ± 0.02	n.d.	n.d.	n.d.	n.d.	n.d.	
Kaempferol-hexoside	1.04 ± 0.09	0.06 ± 0.00	0.07 ± 0.02	0.05 ± 0.00	0.01 ± 0.00	0.01 ± 0.00	
Kaempferol-acetyl-hexosides	0.43 ± 0.05	0.19 ± 0.00	n.d.	n.d.	n.d.	n.d.	
Kaempferol-3-rutinoside	n.d.	n.d.	n.d.	n.d.	0.31 ± 0.04	n.d.	
Kaempferol-3-glucuronide	n.d.	0.19 ± 0.01	1.05 ± 0.07	0.56 ± 0.02	2.95 ± 0.28	0.38 ± 0.01	
Kaempferol-rhamnoside-hexoside	0.10 ± 0.01	n.d.	n.d.	n.d.	0.09 ± 0.01	n.d.	
Kaempferol-galloyl-hexoside	0.22 ± 0.02	n.d.	n.d.	n.d.	0.02 ± 0.00	n.d.	
Kaempferol-glucuronyl-hexoside	n.d.	n.d.	n.d.	n.d.	0.02 ± 0.00	n.d.	***
Isorhamnetin hexoside	n.d.	0.19 ± 0.01	n.d.	1.34 ± 0.07	n.d.	n.d.	
Isorhamnetin acetyl hexoside	n.d.	0.31 ± 0.01	n.d.	n.d.	n.d.	n.d.	
Isorhamnetin-3-rutinoside	n.d.	0.03 ± 0.00	n.d.	n.d.	n.d.	n.d.	***
Myricetin-3-glucuronide	n.d.	n.d.	n.d.	n.d.	0.17 ± 0.03	n.d.	
Myricetin hexoside	n.d.	n.d.	n.d.	0.64 ± 0.02	n.d.	n.d.	
Laricitrin-3-glucuronide	n.d.	0.57 ± 0.01	n.d.	0.23 ± 0.01	n.d.	n.d.	
Laricitrin	n.d.	n.d.	n.d.	n.d.	n.d.	0.65 ± 0.03	
Syringetin	n.d.	n.d.	n.d.	n.d.	n.d.	0.44 ± 0.03	
**Flavonols**	**5.97 ± 0.58 ^a^**	**4.05 ± 0.14 ^abc^**	**1.45 ± 0.14 ^c^**	**5.07 ± 0.28 ^ab^**	**4.07 ± 0.49 ^abc^**	**3.23 ± 0.10 ^bc^**	***
Naringenin-hexosides	n.d.	n.d.	0.17 ± 0.05 ^b^	n.d.	32.07 ± 2.05	n.d.	***
**Flavanones**	n.d.	n.d.	**0.17 ± 0.05 ^b^**	n.d.	**32.07 ± 2.05**	n.d.	***
Vescalagin	6.55 ± 1.27	n.d.	n.d.	n.d.	n.d.	n.d.	***
**Ellagitannins**	**6.55 ± 1.27**	**n.d.**	**n.d.**	**n.d.**	**n.d.**	**n.d.**	
Gallic acid	n.d.	0.35 ± 0.01 ^b^	n.d.	n.d.	n.d.	n.d.	
Digalloyl-HHDP-hexoside isomer	2.39 ± 0.52 ^a^	n.d^.^	n.d.	n.d.	n.d.	n.d.	***
**Gallotannins**	**2.39 ± 0.52 ^a^**	**0.35 ± 0.01 ^b^**	n.d.	n.d.	n.d.	n.d.	***
**TOTAL**	**41.70 ± 6.59 ^ab^**	**15.00 ± 0.42 ^b^**	**13.00 ± 2.90 ^b^**	**67.78 ± 0.57 ^a^**	**45.50 ± 4.32 ^ab^**	**29.02 ± 0.65 ^ab^**	

Different letters (^a–c^) in the same row indicate significant differences, determined by LSD range test, in phenolic content between different invasive plant species; n.d.—not detected. (*–statistically significant differences at *p*-value < 0.05, **–statistically significant differences at *p*-value < 0.001, ***–statistically significant differences at *p*-value < 0.0001.)

**Table 2:** Yield of extract (mg/g), cytotoxicity (LC_50_) against Vero African green monkey kidney cells (mg/mL), minimum inhibitory concentration (MIC; mg/mL), total antibacterial activity (TAA; yield/MIC) after 24 h incubation (mL/g), and selectivity indices (SI; LC_50_/MIC) of the acetone extracts of different plant species and gentamicin (positive control) against test bacteria.

Plant Species	Yield	LC_50_	*Escherichia coli*			*Enterococcus faecalis*			*Staphylococcus aureus*			*Pseudomonas aeruginosa*			Average		
			MIC	TAA	SI	MIC	TAA	SI	MIC	TAA	SI	MIC	TAA	SI	MIC	TAA	SI
*A. altissima*	42	0.17	0.23	183	0.74	0.04	1050	4.25	0.31	135	0.55	0.31	135	0.55	0.22	376	1.52
*A. artemisiifolia*	101	0.02	0.31	326	0.06	0.06	1683	0.33	0.31	326	0.06	0.63	160	0.03	0.32	624	0.12
*C. canadensis*	47	0.17	0.31	152	0.55	0.04	1175	4.25	1.88	25	0.09	1.25	38	0.14	0.87	348	1.29
*D. viscosa*	63	0.03	0.16	394	0.19	0.04	1575	0.75	0.31	203	0.10	0.31	203	0.10	0.21	594	0.33
*E. annuus*	66	0.15	0.31	213	0.48	0.04	1650	3.75	1.25	53	0.12	1.25	53	0.12	0.71	532	1.17
*X. strumarium*	57	0.001	0.23	248	0.004	0.04	1425	0.03	0.08	713	0.01	0.08	713	0.01	0.11	775	0.01
Average			0.26	253	0.34	0.04	1426	2.23	0.69	243	0.16	0.64	217	0.16			
Gentamicin			8 × 10^−4^			2 × 10^−4^			1.3 × 10^−4^			3 × 10^−4^			3.6 × 10^−4^		
Doxorubicin		0.012															

**Table 3:** Yield of extract (mg/g), cytotoxicity (LC_50_) against Vero African green monkey kidney cells (mg/mL), minimum inhibitory concentration (MIC; mg/mL), total antifungal activity (TAA; yield/MIC) after 24 h incubation (ml/g), and selectivity indices (SI; LC_50_/MIC) of the acetone extracts of different plant species and amphotericin B (positive control) against test fungi.

Plant Species	Yield	LC_50_	*Candida albicans*			*Cryptococcus neoformans*			*Aspergillus fumigatus*			Average		
			MIC	TAA	SI	MIC	TAA	SI	MIC	TAA	SI	MIC	TAA	SI
*A. altissima*	42	0.17	0.16	269	1.12	0.31	134	0.56	0.16	269	1.12	0.21	224	0.93
*A. artemisiifolia*	101	0.02	0.16	646	0.11	0.63	162	0.03	1.25	81	0.01	0.68	296	0.05
*C. canadensis*	47	0.17	0.16	301	1.09	0.31	150	0.55	0.63	75	0.27	0.37	175	0.64
*D. viscosa*	63	0.03	0.16	403	0.22	0.31	202	0.11	0.63	101	0.05	0.37	235	0.13
*E. annuus*	66	0.15	0.31	211	0.46	0.31	211	0.46	1.25	53	0.12	0.62	158	0.35
*X. strumarium*	57	0.001	0.23	243	0.003	0.16	365	0.01	0.23	243	0.003	0.21	284	0.003
Average			0.20	346	0.50	0.34	204	0.29	0.69	137	0.26			
Amphotericin B			8 × 10^−3^			8 × 10^−3^			8 × 10^−3^					

## Data Availability

Not applicable.

## Supplementary Materials

The following are available online at https://www.mdpi.com/article/10.3390/plants11050596/s1: Figure S1: Heat map presenting the representations of phenolic groups in different invasive plants; Figure S2: Chromatogram of the acetone extracts of the plant species leaves developed in ethyl acetate/methanol/water (EMW) solvent system sprayed with vanillin–sulphuric acid and TLC bioautograms; Table S1: Spectrum, mass-to-charge ratio (m/z) values of the molecular masses, and main fragments (MS2—second-generation product ion, MS3—third-generation product ion) in negative ion mode ((M-H)^−^) identified with ESI–MS and the distribution of individual compounds in different invasive plants.
