# Supplementary material for: LC–DAD–MS Phenolic Characterisation of Six Invasive Plant Species in Croatia and Determination of Their Antimicrobial and Cytotoxic Activity
Source: Plants (Basel). 2022 Feb 23;11(5):596. doi: 10.3390/plants11050596 (PMC8912889; doi:10.3390/plants11050596)
Supplement: Supplementary file 1 [file plants-11-00596-s001.zip › SUPPLEMENTARY MATERIAL-1581673/Figure S1.pdf]

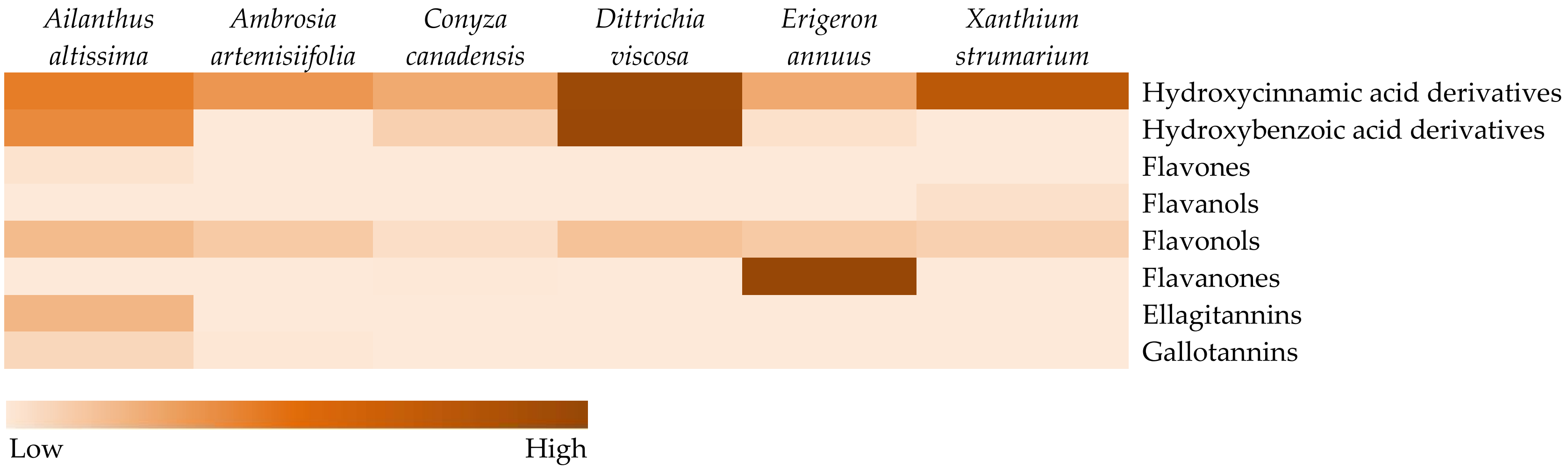

Figure S1. Heat map presenting the representation of phenolic groups in different invasive plants.
