# Supplementary material for: LC–DAD–MS Phenolic Characterisation of Six Invasive Plant Species in Croatia and Determination of Their Antimicrobial and Cytotoxic Activity
Source: Plants (Basel). 2022 Feb 23;11(5):596. doi: 10.3390/plants11050596 (PMC8912889; doi:10.3390/plants11050596)
Supplement: Supplementary file 1 [file plants-11-00596-s001.zip › SUPPLEMENTARY MATERIAL-1581673/Figure S2.pdf]

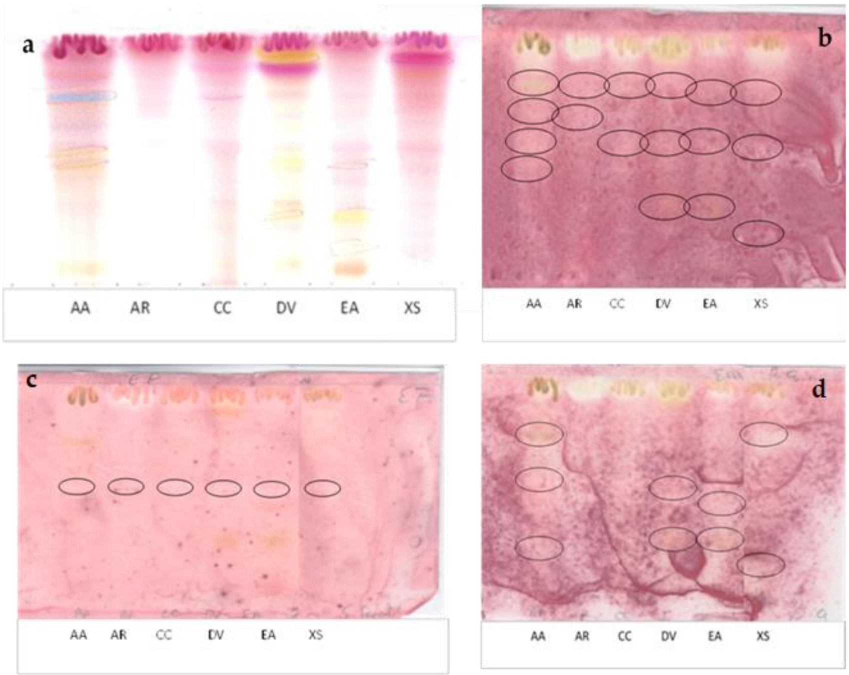

Figure S2. a) Chromatogram of the acetone extracts of the plant species leaves developed in ethyl acetate:methanol:water (EMW) solvent system sprayed with vanillin-sulphuric acid. TLC bioautograms of **b)** *Staphylococcus aureus*, **c)** *Enterococcus faecalis*, and **d)** *Pseudomonas aeruginosa* developed with EMW solvent system. White bands (circled) indicate compounds that inhibit the growth of the bacteria. AA = *A. altissima*; AR = *A. artemisiifolia*; CC = *C. canadensis*; DV = *D. viscosa*; EA = *E. annuus*; XS = *X. strumarium.*
