# Supplementary material for: LC–DAD–MS Phenolic Characterisation of Six Invasive Plant Species in Croatia and Determination of Their Antimicrobial and Cytotoxic Activity
Source: Plants (Basel). 2022 Feb 23;11(5):596. doi: 10.3390/plants11050596 (PMC8912889; doi:10.3390/plants11050596)
Supplement: Supplementary file 1 [file plants-11-00596-s001.zip › SUPPLEMENTARY MATERIAL-1581673/Table S1.pdf]

Table S1. Spectrum, mass to charge ratio (*m/z*) values of the molecular masses and main fragments (MS^2 – second-generation product ion; MS^3 – third-generation product ion) in negative ion mode ($[M\text{-}H]^-$) identified with ESI-MS and the distribution of individual compounds in different invasive plants.

**Phenolics**	λ_{max}	**MS *m/z***	MS^2	MS^3	***Ailanthus altissima***	***Ambrosia artemisiifolia***	***Conyza canadensis***	***Dittrichia viscosa***	***Erigeron annuus***	***Xanthium strumarium***
**Hydroxycinnamic acid derivatives**										
Neochlorogenic acid (3-Caffeoylquinic acid)	234, 326	353	191, 179, 135	173, 127, 85	X	X	X	X	X	X
Cryptogenic acid (4-Caffeoylquinic acid)	234, 328	353	173, 179		X	X	X		X	X
Chlorogenic acid (5-Caffeoylquinic acid)	234, 328	353	191, 179	127, 93, 85	X	X	X	X	X	X
Caftaric acid 1	308, 328	311	179, 149, 135			X				
Caftaric acid 2	308, 328	311	179, 149, 135			X				
Caffeic acid	321	179	135			X				
Caffeic acid derivative 1	272, 325	335	179, 161	135		X			X	
Caffeic acid derivative 2	272, 325	335	179, 161	135		X				
Dicaffeoylquinic acid 1	246, 316	515	353	191, 179		X	X	X	X	X
Dicaffeoylquinic acid 2	246, 316	515	353	191, 179		X	X	X	X	X
Dicaffeoylquinic acid 3	246, 316	515	353	191, 179		X	X	X	X	X
Dicaffeoylquinic acid 4	246, 316	515	353	191, 179				X		
Dicaffeoylquinic acid 5	246, 316	515	353	191, 179				X		
Tricaffeoylquinic acid 1	296, 326	677	497, 515, 353	191, 179					X	X
Tricaffeoylquinic acid 2	296, 326	677	497, 515, 353	191, 179					X	X
3-*p*-Coumaroylquinic acid	312	337	163		X	X	X	X	X	
4-*p*-Coumaroylquinic acid	312	337	173, 163, 155, 137, 191		X					
5-*p*-Coumaroylquinic acid	312	337	191, 173, 163, 155		X	X	X	X	X	X
3-Feruoylquinic acid	320	367	193,134	149	X	X	X			X
5-Feruoylquinic acid	322	367	191	173, 127, 85		X				X
**Hydroxybenzoic acid derivatives**										
Ellagic acid	254,365	301	271, 257, 229, 185				X			
Ellagic acid pentoside	251,360	433	301	299, 257, 185	X					
Ellagic acid hexoside 1	252, 368	463	301	299, 257, 185				X	X	
Ellagic acid hexoside 2	252, 368	463	301	299, 257, 185				X		
Ellagic acid rhamnoside	372, 250	447	301	299, 257, 185					X	

**Flavones**										
Apigenin	268,329	269	225, 183, 151							X
Apigenin hexoside	268,329	431	269	225, 183, 151	X					
**Flavanols**										
Procyanidin dimer	278	577	451,425,407,289							X
Epicatechin	234,279	289	245, 205, 179, 261	203, 227, 161						X
**Flavonols**										
Quercetin-dihexoside	254, 362	625	463	301, 273, 179		X	X		X	
Quercetin galloyl hexoside 1	266, 356	615	463	301, 245, 179	X					
Quercetin galloyl hexoside 2	266, 356	615	463	301, 245, 179	X					
Querctin-3-galactoside	256,356	463	301	179, 151	X	X		X		
Quercetin-3-glucoside	255,355	463	301	179, 151	X	X	X	X	X	X
Quercetin-3-rutinoside	255,355	609	301	179, 151		X		X		X
Quercetin-3-xyloside	256,356	433	301	151		X	X	X		X
Quercetin-3-rhamnoside	266,356	447	301	179					X	
Quercetin glucuronide	256, 354	477	301	179, 151		X	X	X	X	X
Quercetin-3-arabinopyranoside	256, 352	433	301	271, 179		X	X	X		
Quercetin acetyl hexoside	256, 352	549	505	301	X					
Kaempferol hexoside	266,348	447	285	257, 229	X	X	X	X	X	X
Kaempferol acetyl hexoside 1	267, 349	489	429	285	X	X				
Kaempferol acetyl hexoside 2	267, 349	489	429	285	X					
Kaempferol-3-rutinoside	266, 346	593	285						X	
Kaempferol-3-glucuronide 1	348, 266	461	285			X	X	X	X	X
Kaempferol-rhamnoside-hexoside	266, 346	593	285		X				X	
Kaempferol-galloyl-hexoside	348, 266	599	447	313, 285, 169	X				X	
Kaempferol glucuronyl hexoside	348, 266	623	447	285					X	
Isorhamnetin hexoside	255,352	477	357, 315	300, 271, 257		X		X		
Isorhamnetin-acetyl-hexoside	336	519	459, 315	300, 273		X				
Isorhamnetin-3-rutinoside	255, 325	623	315	300, 273		X				
Myricetin-3-glucuronide	261, 349	493	317, 271	171, 151					X	
Myricetin hexoside	261, 349	479	317					X		
Laricitrin-3-glucuronide	253, 354	507	331			X		X		

Laricitrin	265, 372	347	331							X
Syringetin	251, 370	359	345, 315	312, 283						X
**Flavanones**										
Naringenin hexoside 1	283,340	433	271	151			X		X	
Naringenin hexoside 2	283,340	433	271	151					X	
**Ellagitannins**										
Vescalagin isomer	203	953	933, 915, 871, 569	301	X					
**Gallotannins**										
Gallic acid	271	169	125	125		X				
Digalloyl-HHDP-hexoside isomer	262	785	783, 633, 615	483, 301	X					
